# The structural basis of Miranda-mediated Staufen localization during *Drosophila* neuroblast asymmetric division

**DOI:** 10.1038/ncomms9381

**Published:** 2015-10-01

**Authors:** Min Jia, Zelin Shan, Ying Yang, Chunhua Liu, Jianchao Li, Zhen-Ge Luo, Mingjie Zhang, Yu Cai, Wenyu Wen, Wenning Wang

**Affiliations:** 1 Shanghai Key Laboratory of Molecular Catalysis and Innovative Materials, Department of Chemistry and Key Laboratory of Molecular Medicine, Ministry of Education, Institutes of Biomedical Sciences, Shanghai Medical College, Fudan University, Shanghai 200433, China; 2 School of Life Sciences, Fudan University, Shanghai 200433, China; 3 Temasek Life Sciences Laboratory, National University of Singapore, Singapore 117604, Singapore; 4 Department of Biological Sciences, National University of Singapore, Singapore 117543, Singapore; 5 Division of Life Science, State Key Laboratory of Molecular Neuroscience, Hong Kong University of Science and Technology, Clear Water Bay, Kowloon, Hong Kong, China; 6 Institute of Neuroscience and State Key Laboratory of Neuroscience, Shanghai Institutes for Biological Sciences, Chinese Academy of Sciences, Shanghai 200031, China; 7 Department of Systems Biology for Medicine, School of Basic Medical Sciences, Shanghai Medical College of Fudan University, Shanghai 200032, China

## Abstract

During the asymmetric division of *Drosophila* neuroblasts (NBs), the scaffold Miranda (Mira) coordinates the subcellular distribution of cell-fate determinants including Staufen (Stau) and segregates them into the ganglion mother cells (GMCs). Here we show the fifth double-stranded RNA (dsRNA)-binding domain (dsRBD5) of Stau is necessary and sufficient for binding to a coiled-coil region of Mira cargo-binding domain (CBD). The crystal structure of Mira514–595/Stau dsRBD5 complex illustrates that Mira forms an elongated parallel coiled-coil dimer, and two dsRBD5 symmetrically bind to the Mira dimer through their exposed β-sheet faces, revealing a previously unrecognized protein interaction mode for dsRBDs. We further demonstrate that the Mira–Stau dsRBD5 interaction is responsible for the asymmetric localization of Stau during *Drosophila* NB asymmetric divisions. Finally, we find the CBD-mediated dimer assembly is likely a common requirement for Mira to recognize and translocate other cargos including brain tumour (Brat).

## Abstract (web summary)

The scaffold protein Miranda is required for the asymmetric segregation of the RNA binding protein Staufen to ganglion mother cells during *Drosophila* neuroblast division. Jia *et al*. map the interaction between these proteins and present a crystal structure of the interacting domains.

## Graphical abstract

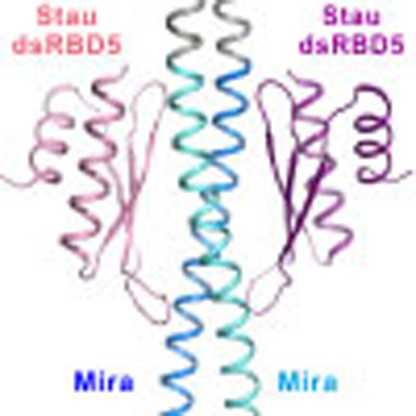

Asymmetric cell division (ACD) is an evolutionarily conserved division mode used by stem and progenitor cells to create two daughter cells with distinct fates^1^,^2^,^3^,^4^. Typically, one daughter cell is a copy of the mother cell retaining self-renewal ability, and the other daughter cell enters the path of differentiation^5^. Much of our understanding of the molecular mechanisms regulating ACD is coming from the studies of NBs, the neural stem cell of *Drosophila* central nervous system^5^. Each ACD of NB generates a self-renewing NB and a smaller GMC that initiates differentiation^6^. During this process, protein complexes containing cell fate determinants localize asymmetrically to the basal cell cortex during mitosis and are segregated preferentially into the GMC daughter after cytokinesis^7^. The cell fate determinants include the transcription factor Prospero (Pros) and its mRNA^8^,^9^,^10^,^11^,^12^,^13^, the posttranscriptional repressor Brat^14^,^15^,^16^, the Notch signalling inhibitor Numb^17^, and the dsRNA-binding protein Stau, which binds the 3′UTR of *pros* mRNA^18^,^19^. The asymmetric localization of these cell fate determinants is facilitated by two adaptor proteins that colocalize with them at the basal cell cortex. While the basal localization of Pros, Brat and Stau is facilitated by their binding to the scaffold protein Mira^16^,^20^,^21^,^22^, the asymmetric localization of Numb is mediated by its adaptor Partner of Numb (Pon)^23^.

Mira is a multi-domain scaffold protein that concentrates at the basal cortex along with its cargos during mitosis of *Drosophila* NB asymmetric division^21^,^22^. After segregating into GMC with its cargo proteins Pros, Brat and Stau, Mira is rapidly degraded^20^,^24^,^25^. The basal localization of Mira in metaphase was proposed to occur upon direct aPKC phosphorylation at the N-terminal cortical localization domain (aa 1–290)^26^. The C-terminal part of Mira was shown to be responsible for cargo release and degradation^27^. The central CBD of Mira (aa 460–668) has been identified as the binding region for cargo proteins Pros, Brat and Stau^14^,^20^,^24^, and was predicted to form a parallel coiled-coil dimer through biochemical and biophysical characterizations^28^. The previously identified binding regions for Pros, Brat and Stau are largely overlapped^14^,^20^,^24^. However, due to the elongated shape of Mira CBD, the actual contact interfaces on Mira should involve much less residues than those of the identified regions. Moreover, it is not clear whether these cargos competitively or simultaneously interact with the scaffold Mira. Therefore, the exact binding sites of the cargo proteins are yet to be determined.

The Stau family of dsRNA-binding proteins exerts a conserved function in mRNA transport, localization and translational control from *Drosophila* to mammals^29^. Stau was originally identified as a factor to anchor *oskar* mRNA and *bicoid* mRNA to the posterior and anterior of the *Drosophila* oocyte, respectively, and is thus required for the correct formation of anteroposterior axis^30^. Its function was then expanded to neurogenesis, as Stau targets *pros* mRNA and facilitates its asymmetric localization and segregation during *Drosophila* NB asymmetric divisions^18^,^19^. Loss of localization of Stau or of *pros* mRNA alters GMC development^18^,^19^. In mammals, two members of the Stau family, STAU1 and STAU2, have been identified. Recent findings that mouse STAU2 regulates *Prox1* (Pros homologue) mRNA and *Trim32* (Brat homologue) mRNA asymmetric localization have demonstrated the conserved role of Stau proteins in neurogenesis^31^,^32^. In addition, both STAU1 and STAU2 are core components in distinct ribonucleoprotein particles for specific dendritic mRNA transport, and therefore play distinct roles in dendrite morphogenesis, synaptic plasticity and memory formation^33^,^34^.

The Stau family use conserved dsRBDs for dsRNA binding, and five dsRBDs have been identified in *Drosophila* Stau (Fig. 1a)^35^. dsRBDs 1, 3 and 4 bind RNA *in vitro*, but dsRBDs 2 and 5 do not^35^. However, both dsRBDs 2 and 5 are required for the anchoring of *bicoid* mRNA, whereas dsRBD5 also directs the actin-dependent localization of *pros* mRNA through interaction with Mira, pointing to other functional roles of dsRBDs 2 and 5 rather than RNA binding^20^,^24^,^25^,^35^. *Drosophila* Stau dsRBDs 2 and 5 belong to the unconventional (type B) dsRBDs, which are incapable of binding dsRNA although may adopt the same α_1_–β_1_–β_2_–β_3_–α_2_ fold as a canonical dsRBD^36^. The function of type B dsRBDs is proposed to mediate protein–protein interactions, although some canonical dsRBDs are recently found to bind proteins as well^36^. However, most dsRBD–protein interactions demonstrated by high-resolution structures are intramolecular (*cis*) interactions, in which appendages N and/or C terminal to the dsRBD pack with the dsRBD α1–α2 helical surface^36^. One example for the few known intermolecular (*trans*) dsRBD–protein interactions is human STAU1 type B dsRBD5, which was shown to mediate dimerization of STAU1 through the interaction between the α1–α2 helical surface of dsRBD5 and the N-terminal appendage Stau-swapping motif (SSM) in a domain-swapped manner^37^. As of yet, all the known *trans*-protein interactions of dsRBDs are dsRBD–dsRBD interactions facilitated by appendages. It is not clear whether and how dsRBDs bind to proteins without appendages. Moreover, as yet there lacks a clear structure illustrating the *trans* dsRBD–protein interactions other than dsRBD–dsRBD dimerization.

Here we perform detailed biochemical characterizations of the interaction between Mira and Stau. The Mira/Stau binding sites are mapped to residues 514–595 of Mira and Stau dsRBD5 (aa 951–1,018). The crystal structure of Mira514–595/Stau dsRBD5 is solved at 2.5 Å. The complex is found to form a 2:2 heterotetramer in which Mira forms an elongated parallel coiled-coil dimer, and two dsRBDs bind to two symmetric surfaces at the N-terminal end of the Mira dimer interface. To the best of our knowledge, this is the first high-resolution complex structure describing the intermolecular dsRBD–protein interaction. Stau dsRBD5 uses the exposed face of its β-sheet to interact with Mira, revealing a novel target binding mode for dsRBDs. We also demonstrate that the direct interaction between Mira and Stau dsRBD5 is crucial for the asymmetric localization of Stau in asymmetrically dividing NBs of *Drosophila* larval brain. We further showed that the dimerization of Mira CBD is essential to coordinate the polarized distribution of its cargos Stau, Brat and Pros *in vivo*.

## Results

### The interaction between Mira and Stau

**Figure 1:**
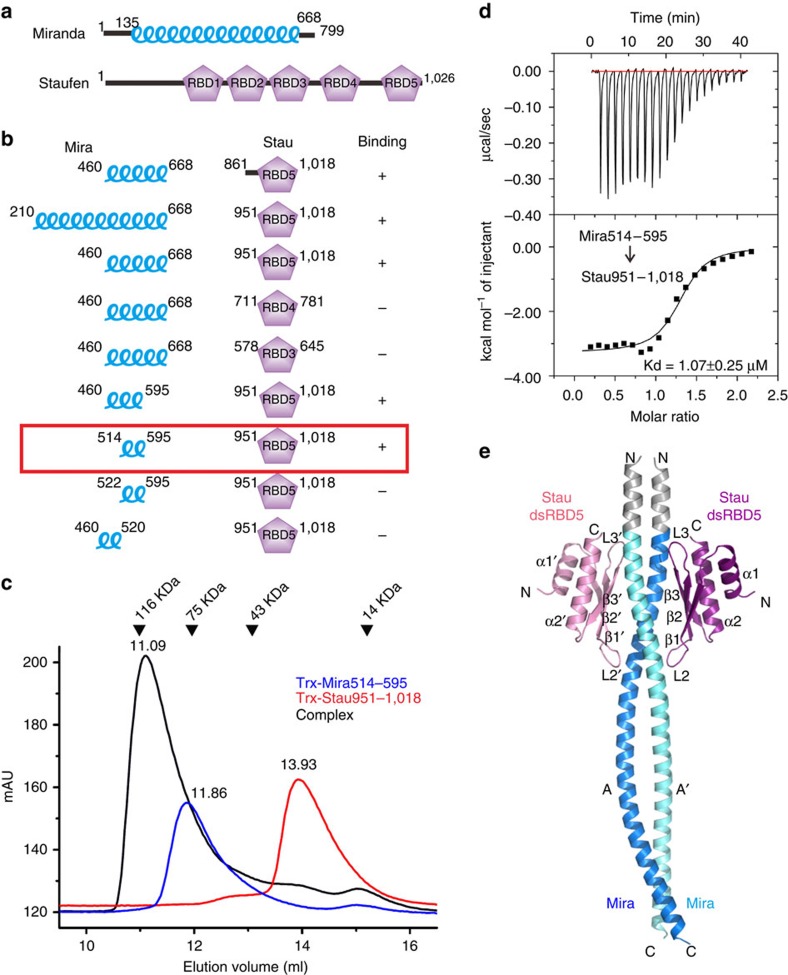
The interaction between Mira and Stau. (**a**) Domain organization of Miranda and Staufen. (**b**) SEC-based analyses of the interactions between various Mira and Stau fragments. (**c**) SEC profiles of Trx-Mira514–595 (blue), Trx-Stau951-1,018 (red) and the Trx-Mira514–595/Trx-Stau951-1,018 complex (black), showing pronounced shift in elution volume for the complex compared with either of the two individual proteins. mAU, milliabsorbance units. The elution volumes of the peaks and the molecular mass standards are indicated at the top of the panel. (**d**) ITC of purified Trx-Mira514–595 and Trx-Stau951-1,018, indicating a complex with a dissociation constant of 1.07±0.25 μM. (**e**) Ribbon diagram representation of the Mira514–595/Stau dsRBD5 complex structure. Mira coiled-coil dimer is coloured in navy blue and cyan, Stau dsRBD5s are coloured in purple and pink, and the grey part indicates the remained fragment after Trx-tag cleavage.

Previous studies reported that Stau interacts with Mira through the C-terminal dsRBD5 domain, and the Stau binding site on Mira was mapped to a segment of residues 506–638 (ref. 20). To verify this interaction, we purified various fragments of these two proteins and performed *in vitro* binding assays. First, we tested bindings of the whole CBD of Mira (aa 460–668) with various fragments of C-terminal region of Stau containing dsRBD5, as well as the dsRBD3 and dsRBD4 (Fig. 1b). The size-exclusion chromatography (SEC) experiments showed that the 68 amino acids fragment of Stau dsRBD5 (aa 951–1,018) is sufficient to form a complex with Mira, while the N-terminal extensions are not necessary (Fig. 1b and Supplementary Fig. 1). The dsRBD3 (aa 578–645) and dsRBD4 (aa 711–781) of Stau, as expected, could not interact with Mira CBD. Then we tested various C-terminal and N-terminal truncations of Mira CBD, and identified that a fragment of amino acids 514–595 in Mira (referred to as Mira514–595 hereafter) is necessary and sufficient to bind Stau dsRBD5 (Fig. 1b,c and Supplementary Fig. 1). Further isothermal titration calorimetry (ITC) experiment demonstrated that Mira514–595 binds to Stau dsRBD5 with a relatively high affinity with a dissociation constant around 1 μM (Fig. 1d).

### Overall structure of the Mira514–595/Stau dsRBD5 complex

To understand the molecular basis underlying the interaction between Mira and Stau, we co-purified Mira514–595 and Stau dsRBD5 (aa 951–1,018), and obtained good diffraction-quality crystals of Mira514–595/Stau dsRBD5 complex. The complex structure was solved by single-wavelength anomalous dispersion using Se–Met derivatives at 2.5 Å resolution (Table 1). The entire lengths of Mira514–595 and Stau dsRBD5 are well resolved, except for the last six residues at the C terminus of Mira514–595. The structure revealed that the complex forms a 2:2 heterotetrameric architecture, in which two Stau dsRBD5s interact symmetrically with the N-terminal end of the Mira coiled-coil homodimer (Fig. 1e and Supplementary Fig. 2a). The 2:2 stoichiometry of the Mira514–595/Stau dsRBD5 complex in solution was further confirmed by the static light-scattering experiment (Supplementary Fig. 1h, blue line). The Stau dsRBD5 adopts the α_1_–β_1_–β_2_–β_3_–α_2_ fold as that of human STAU1 dsRBD5 (ref. 37) and many other canonical dsRBDs^36^,^38^. In accord with the previous small-angle X-ray scattering-based structure prediction^28^, two Mira514–595 fragments, each made up of a single contiguous α-helix, dimerize in a parallel manner to form an elongated ‘rod-like' structure (Fig. 1e). Unexpectedly, the 13-aa fragment ‘GPGSEFELRRQAS' N terminal to Mira514–595 which is remained after tag cleavage also forms an α-helix and thus extends the parallel coiled-coil of Mira. Yet, none of the residues from the N-terminal extension is directly involved in Stau dsRBD binding, and the N-extension of Mira514–595 might somehow stabilize the coiled-coil structure (Fig. 1e).

### The Mira coiled-coil dimer

**Figure 2:**
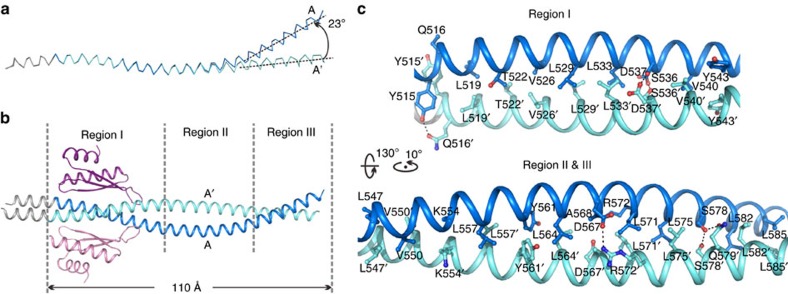
The coiled-coil structure of Mira514–595. (**a**) The Mira514–595 forms an asymmetric coiled-coil with one helix curved about 23 degrees. (**b**) The Mira514–595 coiled-coil contains three potential target-binding regions, with region I interacting with Stau dsRBD5. (**c**) The dimerization interface of the Mira coiled-coil. The side chains of the residues involved in the dimer interface formation are drawn in the stick model. Dotted lines denote hydrogen bonds and salt bridge interactions.

Interestingly, the Mira coiled-coil dimer is slightly asymmetric, with one curved helix A wrapping around the other approximately straight one A′ (Figs 1e and 2a). Two helices of the Mira dimer are identical at the Stau dsRBD5-binding region, spanning a region of ∼30 aa at the very N-terminal end of the Mira514–595 fragment, between Asn514 and Tyr543. Outside this region, helix A kinks about 23 degrees compared with A′ at the C-terminal part of Mira coiled-coil dimer (Fig. 2a). However, the formation of the asymmetric coiled-coil dimer does not disturb the regular heptad repeats (Supplementary Fig. 2b). The entire parallel homodimer in the Mira514–595 structure is ∼110-Å long. On the basis of the analysis of the exposed surfaces of the coiled-coil architecture, we identified three potential target recognition regions in this partial cargo-binding domain of scaffold Mira (Fig. 2b and Supplementary Fig. 2c). Region I encompasses residues 514–543, which is occupied when cargo Stau dsRBD5 is loaded. Region II and III span residues 544–568 and 569–589, respectively.

The two α-helices of Mira514–595 form a quite tight supercoil with an average interhelical distance ∼5.8 Å, a value that is much lower than expected for a canonical coiled-coil (∼9.6 Å)^39^. The tight dimerization of the Mira coiled-coil is mediated by both hydrophobic and polar interactions. The *a*/*d* positions of the two α-helices are mainly hydrophobic residues, which form extensive hydrophobic interactions with each other through their aliphatic side chains (Fig. 2c and Supplementary Fig. 2b). An interhelical salt bridge formed between Asp567 and Arg572′ also contributes to the coiled-coil formation. In addition, several side chain hydrogen bonds formed between two α-helices further stabilize the dimer. Specifically, Tyr515, Ser536 and Asp537 form hydrogen bonds with Gln516′, Asp537′ and Ser536′ from the neighbouring α-helix, respectively. And Ser578 from helix A forms hydrogen bonds with Ser578′ and Gln579′ from helix A′ (Fig. 2c). Despite of the slight asymmetry, surface analysis reveals that all the three potential target-binding surfaces in Mira514–595 dimer are more or less symmetric, indicating that Mira might also interact with other targets in 2:2 stoichiometry through region II and III (Supplementary Fig. 2c). The target recognition of region I and II might be mainly mediated by hydrophobic packing; whereas target binding of region III might be mainly driven by polar interactions.

### The Mira514–595/Stau dsRBD5 interface

**Figure 3:**
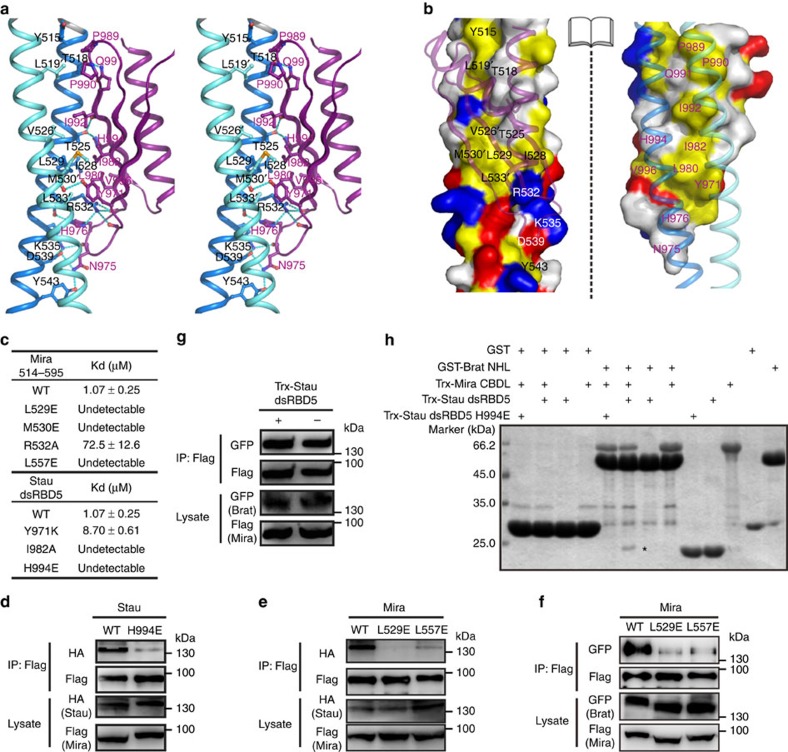
The interface of the Mira514–595/Stau dsRBD5 complex. (**a**) Stereoview showing the interaction details between Mira514–595 and Stau dsRBD5. Dotted lines denote hydrogen bonds and salt bridge interactions. (**b**) Open-book view of the Mira514–595/Stau dsRBD5 complex showing the surface complementation between the coiled-coil dimer and dsRBD5. In this drawing, the hydrophobic residues are coloured in yellow, the positively charged residues in blue, the negatively charged residues in red, and the rest of the amino acids in grey. (**c**) Summary of the bindings between wild type and various Mira514–595 and Stau dsRBD5 mutants derived from ITC analyses. Also see Supplementary Fig. 3. (**d**–**f**) HEK293T cells were transfected with full-length Flag-Mira and either HA-Stau or GFP–Brat. Uncropped blots are shown in Supplementary Fig. 6. (**d**) Mira^WT^ could coimmunoprecipitate with Stau^WT^, but not Stau^H994E^. (**e**) Mira^L529E^ and Mira^L557E^ could not coimmunoprecipitate with Stau^WT^. (**f**) L529E^Mira^ or L557E^Mira^ mutation abolished or significantly impaired the interaction between Mira and Brat. (**g**) Competition assay. HEK293T cells were cotransfected with full-length Flag-Mira and GFP–Brat. Lysates were loaded on the anti-Flag M2 affinity gel, and further incubated with or without 0.8 mg purified Trx-Stau dsRBD5. Presence of an excess amount of Trx-Stau dsRBD5 did not interfere with the interaction between Flag-Mira and GFP–Brat. (**h**) GST Pull down assay. GST-tagged Brat NHL domain could only form a complex with wild-type Stau dsRBD5 (but not the H994E mutant) in the presence of Mira CBDL. Stau dsRBD5 pulled down by GST-tagged Brat NHL via the bridging of Mira CBDL was indicated with a star.

The interesting feature of the Mira514–595/Stau dsRBD5 complex structure is that Stau dsRBD5 binds to Mira through the exposed face of its β-sheet, a mode that has never been observed in any other dsRBDs before this study^36^. Both α-helices of the tightly dimerized Mira coiled-coil are required to form the two identical surfaces at region I for Stau dsRBD5 residing. Structural details at the Mira-dsRBD5 interface show that hydrophobic interactions have significant contributions to the binding (Fig. 3a,b). Hydrophobic residues, such as Tyr971^Stau^ on β1, Leu980^Stau^ and Ile982^Stau^ on β2, Ile992^Stau^ and Val996^Stau^ on β3 of dsRBD5, Ile528^Mira^ and Leu529^Mira^ from helix A, and Val526′^Mira^, Met530′^Mira^, and Leu533′^Mira^ from helix A′ form the hydrophobic core at the interface (Fig. 3a,b). On the other hand, polar interactions are formed across the interface and contribute to the Mira-dsRBD5 binding. For example, the side chain of Thr525^Mira^ from helix A interacts with the main chain oxygen of Ile992^Stau^ and the δ-amide of His994^Stau^ from dsRBD5, respectively (Fig. 3a,b). In addition to the β-sheet, two loop regions (L2 and L3) of Stau dsRBD5 are involved in Mira binding. Arg532^Mira^ from helix A forms an extensive hydrogen bond network with the side chain of His976^Stau^, and main chain carbonyls of Pro972^Stau^ and Glu978^Stau^ at L2 of dsRBD5 (Fig. 3a,b). Next to it, Asn975^Stau^ at the tip of L2 forms hydrogen bonds with Asp539^Mira^ and Tyr543^Mira^ from helix A. Whereas Pro989^Stau^, Pro990^Stau^ at L3 of dsRBD5, Tyr515^Mira^ from helix A, and Leu519′^Mira^ from helix A′ form a hydrophobic core to further stabilize the Mira–Stau interaction (Fig. 3a,b).

### Validation of the Mira–Stau interaction *in vitro*

To verify the Mira–Stau interaction mode observed in the crystal structure, we mutated several residues at the binding interface and subjected the mutant proteins to *in vitro* binding assays. In agreement with the solved crystal structure, SEC and ITC experiments showed that mutations on Mira (L529E, M530E and R532A) or Stau (Y971K, H994E and I982A) from the Mira–Stau packing interface disrupted or severely impaired the interaction between Mira514–595 and dsRBD5 (Fig. 3c, Supplementary Fig. 3). Note that L529 is also located at the interface of Mira coiled-coil dimer (Fig. 2c), and as expected, L529E^Mira^ mutation abolished Mira514–595 dimerization, leading it to elute as a monomer on the SEC profile (Supplementary Fig. 3a). According to the Mira514–595/Stau dsRBD5 complex structure, both α-helices of the Mira coiled-coil dimer are required to interact with Stau dsRBD5 (Fig. 3a). Thus, the effect of L529E^Mira^ mutation might be resulted from direct disruption of the Mira–Stau packing interface and/or breakage of Mira CBD dimer. To verify the importance of intact Mira CBD dimer in Stau binding, we designed the L557E^Mira^ mutation that destroyed the Mira514–595 dimerization but is outside the Stau dsRBD5-binding site (Fig. 2c and Supplementary Fig. 3d). In line with the complex structure, L557E^Mira^ mutation was also found to disrupt the Mira–Stau interaction (Supplementary Fig. 3d,k), demonstrating that the dimer assembly of Mira CBD is crucial for Stau dsRBD5 binding.

Furthermore, the Mira–Stau interaction and the complex structure were validated in the context of full-length proteins by coimmunoprecipitation (Co-IP) experiments. To avoid potentially competing interactions with endogenous proteins expressed in *Drosophila* cell lines, full-length wild type (WT) or mutated Mira was cotransfected with WT or mutant Stau in human HEK293T cells. The Co-IP results demonstrated that the H994E^Stau^, L529E^Mira^ and L557E^Mira^ mutations completely or severely disrupted the Mira–Stau interaction (Fig. 3d,e). Therefore, the binding between Mira and Stau full-length proteins in cell is solely mediated by the interaction between Mira514–595 dimer and Stau dsRBD5. Consistent with previous finding that Brat requires Mira CBD for proper basal localization^14^, the L529E^Mira^ and L557E^Mira^ mutations not only abolished the Mira–Stau interaction, but also significantly attenuated the binding between the full-length Mira and Brat (Fig. 3f), indicating that the compact dimerization of Mira CBD might be a common requirement for cargo recognition through CBD.

### Mira can interact with Stau and Brat simultaneously

As both Stau and Brat bind to Mira CBD^14^,^20^,^24^, we next asked whether Stau and Brat competitively or simultaneously interact with Mira. We first showed that Stau dsRBD5 does not compete with Brat for Mira binding (Fig. 3g). Although Mira CBD (aa 460–668) has been suggested to be essential for Mira–Brat interaction through binding to Brat NHL (NCL-1, HT2A and LIN-41) domain^14^, we found CBD fragments including Mira460–668 and Mira514–595 could not form complex with Brat NHL. Secondary structure prediction using psipred (http://bioinf.cs.ucl.ac.uk/psipred/) reveals that the coiled-coil region of Mira spans a region of ∼530 aa on Mira (aa 135–668; Fig. 1a), pointing to the possibility the previously defined Mira CBD (aa 460–668) may be not long enough for binding to Brat. On the other hand, this data also suggested that the potential cargo-binding Region II and III of Mira514–595 might not be involved in interacting with Brat (Fig. 2b and Supplementary Fig. 2c). Then we made an N-terminal extended Mira CBD (aa 210–668, referred to as Mira CBDL hereafter) and found it was sufficient (maybe not the minimal region) for Brat NHL binding (Fig. 3h). As expected, Mira CBDL robustly interacts with Stau dsRBD5 (Fig. 1b). Importantly, although no direct interaction between Brat NHL and Stau dsRBD5 could be observed, glutathione S-transferase (GST)-tagged Brat NHL could form a complex with wild type Stau dsRBD5 via the bridging of Mira CBDL (Fig. 3h). In contrast, the Mira-binding-deficient H994E mutant could not be pulled down by GST-tagged Brat NHL even in the presence of Mira CBDL, suggesting that Mira may use its elongated coiled-coil structure to simultaneously recruit multiple cargos for transport.

### Mira–Stau binding is essential for Stau localization *in vivo*

We next employed type I NBs in *Drosophila* larval central brain as an *in vivo* model to address whether the Mira–Stau dsRBD5 interaction is of functional significance. To test this, we generated transgenic flies expressing full-length Flag-tagged WT or mutant forms of Mira and Stau, respectively.

**Figure 4:**
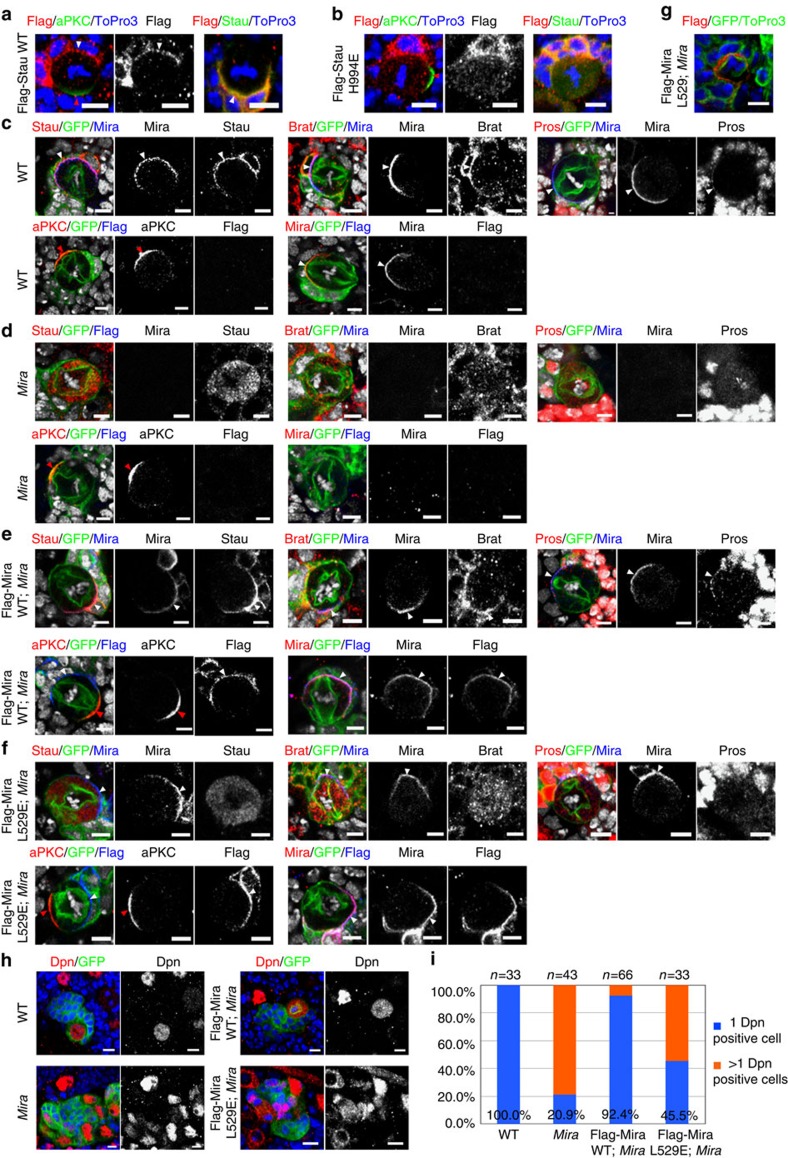
Direct interaction between Mira CBD and Stau dsRBD5 is required for Stau localization during the asymmetric divisions of *Drosophila* type I larval NBs. ToPro3 in blue in **a**, **b**, **g**, **h** and in white in **c**–**f**. (**a**,**b**) Expression of *Flag-Stau WT* and *Flag*-*Stau H994E* transgenes in NB driven by *insc-gal4*. (**a**) Flag**-**Stau WT is localized on the basal cortex in a wild-type NB. (**b**) Flag-Stau H994E shows cytosolic localization in a wild-type NB. (**c**–**h**) NBs are marked by GFP using mosaic analysis with a repressible cell marker (MARCM) technique (see Methods). (**c**–**f**) Staining of various apical and basal proteins (red or blue) and GFP (green) in larval neuroblasts derived from wild type (**c**), *Mira* mutant (**d**), *Mira* mutant expressing a *Flag-Mira WT* transgene (**e**), and *Mira* mutant expressing a *Flag-Mira L529E* transgene (**f**). (**c**) Mira, Stau, Brat, Pros and aPKC are asymmetrically localized in wild type NBs. (**d**) Mira is not detected in *Mira* mutant NBs, whereas Stau, Brat and Pros are cytoplasmic in *Mira* mutant NBs. aPKC is normally localized in a *Mira* mutant NB. (**e**) Mira (detected by anti-Mira antibody or anti-Flag antibody), Stau, Brat, Pros and aPKC are normally localized in *Mira* mutant NBs rescued with Flag-Mira WT. Interestingly, in *Mira* mutant NBs rescued with Flag-Mira L529E, majority of NBs exhibit basal localization of Mira L529E (**f**, detected by anti-Mira antibody or anti-Flag antibody), 5.3% of these NBs showing broad cortical localization of Flag-Mira L529E (**g**). Stau, Brat and Pros remain in cytoplasm and aPKC is normally localized in these NBs. White arrowheads label basal cortex, whereas red arrowheads indicate apical cortex. Scale bar, 5 μm. (**h**) A WT NB lineage contains one Dpn-positive cell, while a *Mira* mutant NB lineage harbours multiple Dpn-positive cells that is reverted by expression of Flag-Mira WT but only partially recused by Flag-Mira L529E variant. (**i**) Statistical data for **h**.

During NB division, Mira is asymmetrically localized on the basal cortex from prophase and is preferentially segregated into GMC daughter on the completion of mitosis. We first examined the localizations of these transgenes in a WT background and focus on mitotic NBs. As expected, Flag-Mira WT (*n*=22, 100%) exhibited basal localization in metaphase NBs (Supplementary Fig. 4a). Interestingly, Flag-Mira L529E (*n*=23, 100%) also localized to the basal cortex (Supplementary Fig. 4b), implying dimerization via Mira CBD is not the only way for Mira basal targeting in a WT background and is consistent with previous finding showing that the N-terminal cortical localization domain (aa 1–290) plays an important role in Mira localization^24^,^25^,^26^,^27^. In line with this, the localization of endogenous apical complex components and basal cell fate determinants in these backgrounds was normal (Supplementary Fig. 4a,b). Predictably, Flag-Stau WT (*n*=14, 100%) also showed the expected basal enrichment in metaphase NBs (Fig. 4a). In contrast, the Mira-binding-deficient Flag-Stau H994E (*n*=21, 100%) could not form the basal crescent but localized into cytoplasm instead (Fig. 4b), supporting our structural analysis and *in vitro* results and demonstrating that the direct interaction between Mira and Stau dsRBD5 is responsible for the correct localization of Stau during ACD.

### Cargo targeting requires intact Mira CBD dimer

We next addressed basal protein localization in *Mira* mutant background rescued with these Mira variants. As reported previously, all Mira cargos including Stau, Brat, and Pros were basally co-localized with Mira in WT NBs (Fig. 4c, Stau, *n*=21, 100%; Brat, *n*=21, 100% and Pros, *n*=28, 100%, also see Supplementary Fig. 4c) but delocalized in *Mira* mutant NBs (Fig. 4d, Stau, *n*=20, 100%; Brat, *n*=20, 100% and Pros, *n*=17, 100%, also see Supplementary Fig. 4d). Expression of Flag-Mira WT in *Mira* mutant NBs fully restored the basal localization of these cargos (Stau (*n*=16, 100%), Brat (*n*=12, 100%), and Pros (*n*=12, 100%)) in dividing NBs (Fig. 4e), also see Supplementary Fig. 4e). Similar to Flag-Mira WT the majority of Flag-Mira L529E (94.7%, *n*=19) also formed basal crescent in *Mira* mutant NBs (Fig. 4f and Supplementary Fig. 4f). Interestingly, 5.3% of NBs examined showed broad cortical localization of Flag-Mira L529E (Fig. 4g). These data suggest that the Mira CBD dimer does play a role in effective basal targeting of Mira. Although Flag-Mira L529E basally localized in *Mira* mutant, endogenous Stau (*n*=26, 100%), Brat (*n*=22, 100%), and Pros (*n*=15, 93.3%) exhibited cytoplasmic localization in this background (Fig. 4f and Supplementary Fig. 4f), pointing to the importance of the intact CBD dimer in the localization of Mira-related basal proteins. As an internal control, aPKC was normally enriched on the apical cortex in *Mira* mutant NBs with or without expression of Flag-Mira variants (Fig. 4c–f; 4d, *n*=16, 100%; 4e, *n*=21, 100%; 4f, *n*=16, 100%; Supplementary Fig. 4c–f).

To further understand the function of Flag-Mira L529E variant, we investigated whether it could rescue *Mira* mutant over-proliferating phenotype. In WT, each WT NB lineage contains one cell expressing NB marker Dpn; however, majority of *Mira* mutant NB lineage overproliferate and contain multiple Dpn-positive cells (Fig. 4h,i). While restoring Flag-Mira WT expression in *Mira* mutant NBs largely reverted the formation of ectopic Dpn-positive cells, ectopic expression of Flag-Mira L529E only partially suppress this phenotype (Fig. 4h,i), suggesting that proper cargo-binding/targeting is important for Mira function. Together with our *in vitro* biochemical analysis, our data clearly demonstrated that the dimerization of Mira CBD is most likely a prerequisite for cargo recognition and translocation.

### Comparison of the Stau dsRBD5 to other dsRBDs

**Figure 5:**
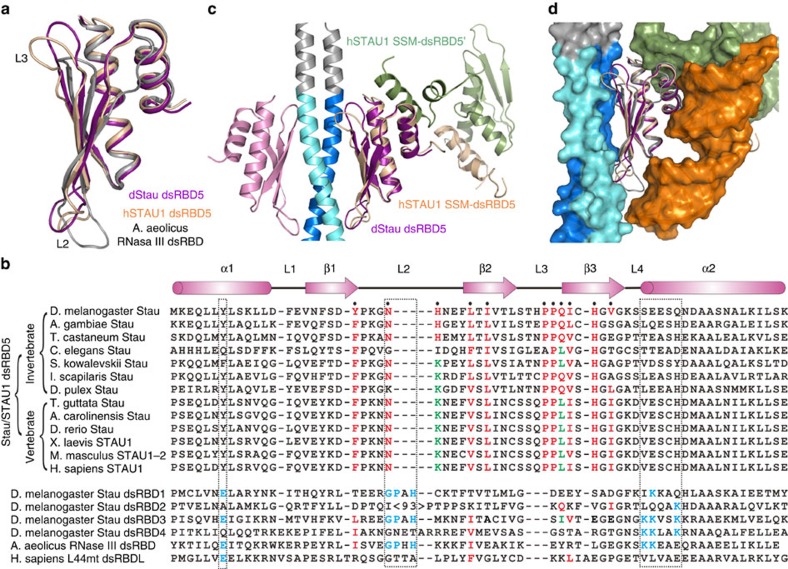
Comparison of the Stau dsRBD5 structure with other dsRBDs. (**a**) Superimposition of Stau dsRBD5 with human STAU1 dsRBD5^37^ (PDB ID: 4DKK) and *A. aeolicus* RNase III dsRBD^40^ (PDB ID: 2NUG). Stau dsRBD5 is coloured in purple, STAU1 dsRBD5 in orange and RNase III dsRBD in grey. (**b**) Structure-based (when known) and sequence-based sequence alignment of *Drosophila* Stau dsRBD5 with other dsRBD5s of Stau/STAU1 homologues (upper part), and with *Drosophila* Stau dsRBD1-4, canonical dsRBD *A. aeolicus* RNase III, and human L44mt dsRBDL^45^ (PDB ID: 3J7Y) (lower part). Secondary structures of *Drosophila* Stau dsRBD5 are shown at the top of the panel. The residues of Stau dsRBD5 involved in Mira binding are indicated with black dots. Conservation of these Mira interacting residues is highlighted. Identical residues or residues with similar polarity or hydrophobicity from invertebrate to vertebrate are coloured in red, those only identical in higher order species in green, and others in black. Three dsRNA-binding regions are indicated by dashed frames. The key residues for dsRNA-binding are highlighted in cyan. (**c**) Superimposition of Mira514–595/Stau dsRBD5 complex and human STAU1 SSM–dsRBD5 dimer structures showing that the β-sheet face mediated protein binding of dsRBD5 does not conflict with the dimerization through the α1–α2 interface. STAU1 SSM–dsRBD5 dimer is coloured in beige and green, Stau dsRBD5s are coloured in purple and pink, and Mira dimer is coloured in navy blue and cyan. (**d**) Superimposition of Stau dsRBD5, human STAU1 dsRBD5 and *A. aeolicus* RNase III dsRBD with Mira (navy blue and cyan), SSM (dark green) and dsRNA (orange) in surface representations, showing that there is no steric hindrance among dsRNA binding and protein binding through β-sheet and α1–α2 interfaces.

The atypical *Drosophila* Stau dsRBD5 adopts a very similar structure as the canonical dsRBD of *Aquifex aeolicus* RNase III (ref. 40), as well as human STAU1 dsRBD5 (ref. 37; Fig. 5a). Similar with its human orthologue, the structure of *Drosophila* Stau dsRBD5 is consistent with its inability of dsRNA binding^35^, lacking the residues important for RNA binding in the canonical dsRBDs, such as *A. aeolicius* RNase III RBD (Fig. 5b). Importantly, the key residues (namely Tyr971, Leu980, Ile982, Ile992, His994 and Val996) on the exposed β-sheet face of Stau dsRBD5 responsible for Mira interaction are evolutionarily conserved from invertebrate to vertebrate in dsRBD5s of Stau/STAU1 homologues. It is most likely that dsRBD5s in other Stau/STAU1 homologues can also use their exposed β-sheet faces to interact with yet unknown proteins via hydrophobic interactions as the main driving force. On the contrary, sequence identities at the β-sheet side of the canonical dsRBD *A. aeolicius* RNase III and *Drosophila* Stau dsRBD1-4 (refs 35, 41) are quite low (Fig. 5b). For instance, Ile982 in *Drosophila* Stau dsRBD5 is negatively charged in *A. aeolicius* RNase III RBD and *Drosophila* Stau dsRBD4, and changed to alanine in *Drosophila* Stau dsRBD3. Note that our mutagenesis experiment showed that the I982A mutation disrupts the Stau dsRBD5/Mira interaction (Fig. 3c and Supplementary Fig. 3e,k). Accordingly, the substitution of Ile982 to negatively charged glutamic acid is expected to disrupt the Mira–Stau interaction. Even in *Drosophila* Stau dsRBD2, which is incapable of RNA binding, the key residues on the exposed β-sheet face are not conserved (Fig. 5b). Nevertheless, these data are consistent with previous findings that only dsRBD5 is responsible for mediating Mira–Stau interaction^20^.

The most prominent structural differences between *Drosophila* Stau dsRBD5 and human STAU1 dsRBD5 locate at the L2 and L3 loops (Fig. 5a). The crystal structure of the Mira514–595/Stau dsRBD5 complex shows that both loops are involved in the interaction with Mira (Figs 1e and 3a). It is noticeable that the sequence and length of L2 and L3 loops are highly variable in different dsRBDs (Fig. 5b). The large (∼100 aa) insertion of L2 in *Drosophila* Stau dsRBD2 was suggested to preclude dsRNA binding, but to contribute to microtubule-dependent mRNA localization^35^. Therefore, the conformational flexibility of the two loops might facilitate the protein interactions of dsRBDs.

### dsRBDs might interact with proteins and dsRNA simultaneously

In human STAU1 dsRBD5, the SSM motif interacts with the α1–α2 helical face of another STAU1 dsRBD5 to facilitate the dimerization of hSTAU1 (ref. 37). *Drosophila* Stau dsRBD5, however, lacks the conserved SSM motif crucial for dimerization in vertebrate Stau dsRBD5 (ref. 37). Our biochemical data showed that the isolated *Drosophila* Stau dsRBD5 aggregates to some extent in solution, but does not form homogeneous oligomer state. By binding to Mira, a homogeneous 2:2 complex was formed as shown in the crystal structure and static light-scattering experiment (Supplementary Fig. 1h, blue line). To test if the N-terminal extension can promote dimerization of *Drosophila* Stau dsRBD5, we purified a longer fragment of Stau (aa 861–1,018) by extending the N-terminal boundary of dsRBD5. Isolated Stau861–1,018 also showed heterogeneous oligomer states in solution as dsRBD5, and the stoichiometry of the Mira514–595/Stau861–1,018 complex is between 2:2 and 2:4 (Supplementary Fig. 1h, magenta line). Therefore, unlike human STAU1, the N-terminal extension of dsRBD5 is not sufficient for stable dimerization of Stau. However, partial formation of a 2:4 complex indicates that the interaction with Mira does not prevent Stau dimerization. By comparison of the crystal structures of Mira514–595/Stau dsRBD5 complex and hSTAU1 SSM–dsRBD5 dimer, we can clearly see that the β-sheet face-mediated protein interaction would not sterically hinder the dimerization of hSTAU1 dsRBD5 (Fig. 5c). On the other hand, by further superimposing the *A. aeolicius* RNase III RBD/dsRNA complex structure onto the Stau dsRBD5 and hSTAU1 dsRBD5, we found that the dsRNA binding, the β-sheet face mediated protein binding and the α1–α2 face-mediated dimerization are not mutually exclusive (Fig. 5d). This suggests that a canonical dsRBD may use its different faces to bind to dsRNA and proteins simultaneously.

## Discussion

Mira is a key adaptor protein that directs several cell fate determinants to basal cortex during the asymmetric division of *Drosophila* NB. The detailed molecular mechanism and the direct interactions between Mira and its cargos, however, have not been characterized biochemically. Here we characterize the interaction between Mira and dsRNA-binding protein Stau. The crystal structure of the Mira514–595/Stau dsRBD5 complex demonstrates that Mira forms a parallel coiled-coil dimer and two molecules of Stau dsRBD5 symmetrically bind to Mira514–595 through their exposed β-sheet faces. Structural based point mutations (H994E^Stau^ and L529E^Mira^) verified that the direct interaction between Mira and Stau dsRBD5. Two lines of evidence support the functional importance of this interaction. First, the Mira-binding-deficient Stau mutant H994E, when ectopically expressed in NB, exhibited a diffused subcellular distribution during NB ACD. Second, endogenous Stau is defectively localized in *Mira* mutant NB expressing Mira L529E, a Mira variant that does not bind Stau.

The finding that Mira514–595 forms an elongated parallel coiled-coil dimer suggests that the full-length Mira most likely forms a stable homodimer. Moreover, the compact dimerization of Mira (at least the CBD region) seems to be crucial for cargo binding and transport, as the Mira^L529E^ mutant with impaired CBD dimer assembly could not rescue the localization of Mira-dependant basal proteins in *Mira* mutant larval NBs (Fig. 4f), and only partially rescue the over-proliferation phenotype observed in *Mira* mutant brains (Fig. 4h,i). It further points to the possibility that destabilization and unwinding of the Mira CBD coiled-coil through protein modifications (for example, phosphorylation) could be a mechanism for cargo release, which is the final step of Mira-mediated protein transport. On the other hand, the observation that Stau and Brat do not compete with each other for Mira binding (Fig. 3f,g) indicates that Mira acts as an elongated scaffold, which can simultaneously recognize multiple cargos.

Another key finding of this study is that Stau dsRBD5, which belongs to the degenerate type B dsRBDs lacking the dsRNA-binding feature^36^, interacts with proteins through its exposed β-sheet face. The type B dsRBDs are proposed to be important for protein–protein interactions^36^. Taking the Stau family as an example, the RNA-binding deficient dsRBDs 2 and 5 of Stau are essential for the localization of mRNAs encoding cell fate determinants in *Drosophila* eggs and NBs^20^,^24^,^25^,^35^. Similarly, human STAU1 interacts with the 60S subunit of cytoplasmic ribosomes independent of dsRBDs 3 and 4 that bind dsRNA^42^,^43^,^44^. Previous structural studies revealed that the dsRBD–protein interaction often requires N- or C-terminal appendages, which interact with the dsRBD α_1_–α_2_ interface mostly in *cis*, and the known *trans*-protein interactions of dsRBDs are dsRBD–dsRBD interactions facilitated by appendages, such as the human STAU1 dsRBD5 homodimer^36^. The crystal structure of Mira514–595/Stau dsRBD5 complex solved here is the first high-resolution structure illustrating the *trans* dsRBD–protein interaction. It clearly demonstrates that dsRBDs can bind proteins without N- or C-terminal appendages. Thereby, dsRBD itself can serve as an independent protein–protein interaction module. Furthermore, dsRBD fold can also use the β-sheet face to interact with proteins, revealing a novel protein interaction mode of dsRBDs so far. We have identified a few residues on the β-sheet face important for Mira binding, although these residues are only conserved among Stau/STAU1 dsRBD5 homologues (Fig. 5b). Whether the conserved β-sheet of STAU1 dsRBD5 is indeed important for mediating protein interactions with physiological relevance, for example, for ribonucleoprotein particle formation, is an interesting prospect that warrants further investigation.

While this manuscript was in preparation, Brown *et al*. provided the 3.4 Å resolution cryoelectron microscopy structure of the large ribosomal subunit from human mitochondria^45^. Surprisingly, we found that a fragment of the RNase III-like L44mt (protein L44 of the mitochondrial 39S ribosomal subunit) adopts a dsRBD-like (referred to as dsRBDL hereafter) fold, and might bind to ribosomal proteins but not RNA through the exposed β-sheet face and L2 loop (Supplementary Fig. 5a). Even though it is not clear whether the β-sheet face (or together with L2) of L44mt dsRBDL is sufficient to mediate specific protein interactions in the isolated state rather than in a large complex with multiple components, it suggests that the β-sheet face might be a common site to mediate dsRBD–protein interaction. Interestingly, although key residues on the exposed β-sheet faces from L44mt dsRBDL and Stau dsRBD5 share little similarity, both of them are capable of protein binding (Fig. 5b and Supplementary Fig. 5a,b). Specifically, six residues (Tyr971, Leu980, Ile982, Ile992, His994 and Val996) from the β-sheet face of Stau dsRBD5 form a hydrophobic surface to interact with Mira. Whereas in L44mt dsRBDL, only two of the above six residues are hydrophobic (Phe271 and Leu280, corresponding to Leu980 and Ile992 in Stau dsRBD5). Nevertheless, L44mt dsRBDL utilizes its exposed β-sheet face to form two protein-binding patches that might interact with two ribosomal proteins simultaneously. Further structure-based sequence alignment of other canonical or type B dsRBDs and dsRBDLs with known structures revealed that residues from the exposed β-sheet faces are highly variable (Supplementary Fig. 5c). Together with the finding that L44mt dsRBDL might use a flexible β-sheet face (compared with that of Stau dsRBD5) to interact with ribosomal proteins, our results imply that dsRBDs may function as versatile protein-binding domains through their exposed β-sheet faces. Moreover, structural comparison shows that protein binding at the β-sheet face does not sterically hinder the dsRNA binding or the interaction at the α_1_–α_2_ interface (Fig. 5d). Other multifunctional dsRBDs are expected to exist, and these dsRBDs may be capable of simultaneously interacting with dsRNA and proteins.

## Methods

### Protein preparation

Various *Drosophila* Stau and Mira fragments (Fig. 1b), and mutants (Fig. 3c), Brat NHL (aa 756–1,037) were individually cloned into a modified version of pET32a vector in which the thrombin cutting site was replaced by a protease 3C cutting site, and the S-tag was removed. Each of the resulting proteins contained a Trx tag in its N termini. All the mutations were generated using the standard PCR-based method (Supplementary Table 1) and confirmed by DNA sequencing. Recombinant proteins were expressed in *Escherichia coli* BL21 (DE3) host cells at 16 °C and were purified by using a Ni^2+^–NTA agarose affinity chromatography followed by SEC. The N-terminal Trx-tagged fragments of Mira and Stau were cleaved by digesting fusion proteins with protease 3C, and the proteins were purified by another step of SEC.

### Size-exclusion chromatography

SEC experiments were carried out on an AKTA FPLC system (GE Healthcare). Proteins at concentrations of 10–20 μM in a volume of 100 μl were loaded on a Superose 12 10/300 GL column 20 (GE Healthcare) equilibrated with the buffer containing 50 mM Tris (pH 8.0), 100 mM NaCl, 1 mM DTT and 1 mM EDTA. Protein elution was detected by absorbance at 280 nm.

### Isothermal titration calorimetry

ITC measurements were performed on an ITC200 Micro calorimeter (MicroCal) at 25 °C. All protein samples were in 50 mM Tris (pH 8.0), 100 mM NaCl, and 1 mM EDTA buffer. The titrations were carried out by injecting 40 μl aliquots of the Mira fragments into Stau fragments at time intervals of 2 min to ensure that the titration peak returned to the baseline. The titration data were analysed using the program Origin7.0 from MicroCal.

### Crystallography

Crystals of the Se–Met derivative of Mira514–595/Stau dsRBD5 protein complex were obtained by the hanging drop vapor diffusion method at 16 °C. The optimized Mira/Stau complex crystals were grown by mixing 1 μl Mira/Stau complex solution, 0.8μl reservoir solution containing 0.1 M sodium acetate (pH 4.8), 0.7 M 1,6-hexanediol, 0.02 M calcium chloride and 0.2 μl 0.1 M magnesium chloride. Crystals were soaked in crystallization solution containing 20% glycerol for cryoprotection. Se-SAD data were collected at SSRF (Shanghai Synchrotron Radiation Facility in China) beamline BL17U at wavelength of 0.9792 Å. Data were indexed, integrated and scaled using HKL2000. Selenium atoms were initially located by SHELXC/D. Experimental phasing, density modification and automatic model building were performed by AutoSol of PHENIX^46^. The initial model was further rebuilt and adjusted manually with COOT^47^ and was refined by phenix.refine program of PHENIX^46^. The final model has 99% of the residues in the favoured region of the Ramachandran plot with no outliers. The final refinement statistics are summarized in Table 1.

### Coimmunoprecipitations and immunoblotting

Human HEK293T Cells were transiently cotransfected with the full-length Flag-Mira and HA-Stau or green fluorescent protein (GFP)–Brat WT proteins or various mutants using polyethylenimine transfection reagent. For the transient transfections, 6 μg Mira^WT^, Mira^L529E^ or 8 μg Mira^L557E^ were cotransfected with 20 μg Stau^WT^ or 13 μg Stau^H994E^, respectively; and 4 μg Mira^WT^, or 20 μg Mira^L529E^, Mira^L557E^ were cotransfected with 4 μg Brat, respectively. Cells were collected 24 h post transfection and lysed in a buffer containing 50 mM Tris (PH 7.4), 150 mM sodium chlorate, 0.5% Nonidet P-40, 10 mM sodium fluoride, 1 mM Sodium metavanadate, 1 mM DTT, 10 mM PMSF and protease inhibitors. Each lysate was incubated with anti-Flag M2 affinity gel (Sigma) overnight. For the competition assay, the lysate containing cotransfected full-length Flag-Mira and GFP–Brat was incubated with anti-flag M2 affinity gel overnight in the presence or absence of 0.8 mg purified Trx-Stau dsRBD5.

After extensive wash of the beads with the lysis buffer, the captured proteins were boiled in SDS–PAGE loading buffer and subjected to SDS–PAGE. Proteins were transferred to 0.45 μM nitrocellulose membrane (Millipore), and the nitrocellulose membrane was blocked using 3% BSA in TBST (20 mM Tris-HCl (pH 7.4), 137 mM NaCl and 0.1% Tween-20) buffer at room temperature for 1 h, followed by incubation with the following antibodies: Flag (ABclonal), HA (ABclonal), GFP (ABclonal) at 1/2,000 dilution at 4 °C overnight. Membranes were washed three times with TBST buffer, incubated with horseradish peroxidase (HRP)-conjugated Goat anti-Rabbit or anti-Mouse antibody (ABclonal) and visualized on a LAS3000 Chemiluminescent Imaging System.

### GST pull-down assay

For GST pull-down assay, GST or GST-tagged Brat NHL (8 μM for the final concentration) were first loaded to 40 μl GSH-Sepharose 4B slurry beads in a 500-μl assay buffer containing 50 mM Tris (pH 8.0), 100 mM NaCl, 1 mM DTT and 1 mM EDTA. The GST fusion protein loaded beads were then mixed with potential binding partners (24 μM each for the final concentration), and the mixtures were incubated for 1 h at 4 °C. After four times washing, proteins captured by affinity beads were eluted by boiling, resolved by 15% SDS–PAGE, and detected by Coomassie blue staining.

### *Drosophila* S2 cell culture

Mira (Flag-Mira WT and Flag-Mira L529E) and Stau (Flag-Stau WT and Flag-Stau H994E) variants were subcloned into UASt.attB vector (A gift from Konrad Basler).

*Drosophila* S2 cells were grown at 25 °C in Schneider's medium (Invitrogen) supplemented with 10% fetal bovine serum. All transfections were performed using Effectene Reagent (Qiagen) according to the manufacturer's instructions. Briefly, S2 cells were cotransfected with 0.5 μg plasmids of interest (Mira or Stau) together with act-gal4 plasmid. Cells were collected at 48 h later and lysed in Nonidet P-40 lysis buffer containing 50 mM Tris, pH 8.0, 250 mM NaCl, 0.5% Nonidet P-40, 0.2 mM EDTA, protease inhibitor cocktail (complete, Roche) and phosphatase inhibitor. The lysate was collected and cleared by centrifugation at 13,000 r.p.m. for 5 min at 4 °C. The samples were separated by 10% polyacrylamide SDS–PAGE gels followed by transferring to PVDF membranes (Millipore). Mouse anti-Flag antibody (Sigma, 1/2,000), Rabbit anti-Mira antibody (Generated in our lab, 1/1,000) and Rabbit anti-Stau antibody (Daniel St Johnston, 1/1,000) were diluted in TBST with 5% non-fat dry milk.

### Fly genetics

Information about the fly stains used in this study was described in the text or FlyBase (www.flybase.org). Stocks (unless stated below) were obtained from Blooming Stock Center and crosses were maintained at 25 °C on standard medium. Stocks used were *FRT82B*, *elav-gal4*, *insc-gal4*, *Tub-gal80*, *hs-flp*, *UAS-CD8::GFP*, *Miranda[L44]* (Fumio Matsuzaki), *UAS-Flag-Mira WT, UAS-Flag-Mira L529E* and *UAS-Flag-Stau WT* and *UAS-Flag-Stau H994E* were generated in this study.

Mira (Flag-Mira WT and Flag-Mira L529E) and Stau (Flag-Stau WT and Flag-Stau H994E) variants were subcloned into UASt.attB vector (A gift from Konrad Basler) and transgenic lines were generated by BestGene, Inc. (ChinoHills,CA) using *attP* landing site on II chromosome (Best Gene line 9723).

To address these Mira variant localization and function in *Mira* mutant background, these transgenes were subsequently crossed into *FRT82B* or *FRT82B.Mira[L44]* background to obtain following stocks.

UASt–Flag-Mira WT/Sm6.cyo; FRT82B/Tm3.tb

UASt–Flag-Mira WT/Sm6.cyo; FRT82B.Mira[L44]/Tm3.tb

UASt–Flag-Mira L529E/Sm6.cyo; FRT82B/Tm3.tb

UASt–Flag-Mira L529E/Sm6.cyo; FRT82B.Mira[L44]/Tm3.tb

Mosaic analysis with a repressible cell marker technique was used to positively mark mutant clones with a GFP signal according to published protocol^48^. In brief, embryos were collected over a period of 6 h, and larvae (24 h after larval hatching, ALH) was subjected to 1-h heat-shocked treatment at 37 °C, and larvae with desired genotypes were dissected and examined.

### Immunohistochemistry and imaging

Larvae of desired genotype were dissected at 96 h ALH and brains were fixed for 15 min in 3.7% formaldehyde in PBS with 0.1% Triton-X, and later processed for immunochemistry analysis. The following antibodies were used: mouse anti-Mira (Fumio Matsuzaki), 1/30; mouse anti-flag (Sigma), 1/2,000; rabbit anti-Mira^49^ (generated in our lab), 1/1,000; rabbit anti-Stau (Daniel St Johnston), 1/2,000; rat anti-Brat^50^ (Yongqing Zhang), 1/500; mouse anti-Pros (DSHB), 1/20; guinea-pig anti-Dpn (generated in our lab), 1/1,000; rabbit anti-Pon (generated in our lab), 1/2,000; rabbit anti-aPKCζ C20 (Santa Cruz Biotechnologies), 1/1,000; chicken anti-GFP (Abcam), 1/5,000. Secondary antibodies were conjugated to Alexa Fluor 488, Alexa Fluor 555, or Alexa Fluor 633 (Molecular Probes), and used at 1/500, 1/1,000, and 1/250, respectively. TO-PRO-3 (Invitrogen) was used at 1/5,000 for DNA staining and samples were mounted in Vectashield (Vector Laboratories). Images were obtained using Zeiss LSM 510 upright or Leica SP II upright microscope and processed in Adobe Photoshop CS6 and Adobe Illustrator CS6.

## Additional information

**Accession codes:** The atomic coordinates of Mira514–595/Stau dsRBD5 have been deposited to the Protein Data Bank under the accession code 5CFF.

**How to cite this article:** Jia, M. *et al*. The structural basis of Miranda-mediated Staufen localization during *Drosophila* neuroblast asymmetric division. *Nat. Commun.* 6:8381 doi: 10.1038/ncomms9381 (2015).

## Supplementary Material

Supplementary Information Supplementary Figures 1-6, Supplementary Table 1

## Figures and Tables

**Table 1:** Data collection and refinement statistics.

	**Se**–**Met**
*Data collection*	
Space group	*C121*
Cell dimensions	
*a*, *b*, *c* (Å)	200.023, 51.332, 100.274
α, β, γ (°)	90.000, 90.555, 90.000
Wavelength (Å)	0.9792
Resolution (Å)	50.00-2.50 (2.54–2.50)*
*R*_merge_ (%)	7.8 (66.4)
Mean *I*/σ*I*	35.4 (3.7)
Completeness (%)	94.9 (99.8)
Redundancy	5.5 (6.1)
	
*Refinement*	
Resolution (Å)	27.66–2.50
No. of reflections	33,794
*R*_work_/*R*_free_	24.2/28.1
No. atoms	
Protein	4,605
Water	27
B factors	
Protein	42.63
Water	33.19
r.m.s. deviations	
Bond lengths (Å)	0.010
Bond angles (°)	1.183

Abbreviations; r.m.s., root-mean square.

^*^Values in parenthese indicate the highest-resolution shell.
