# Supplementary material for: The structural basis of Miranda-mediated Staufen localization during Drosophila neuroblast asymmetric division
Source: Nat Commun. 2015 Oct 1;6:8381. doi: 10.1038/ncomms9381 (PMC4600727; doi:10.1038/ncomms9381)
Supplement: Supplementary Information — Supplementary Figures 1-6, Supplementary Table 1 [file ncomms9381-s1.pdf]

## Supplementary Figure 1

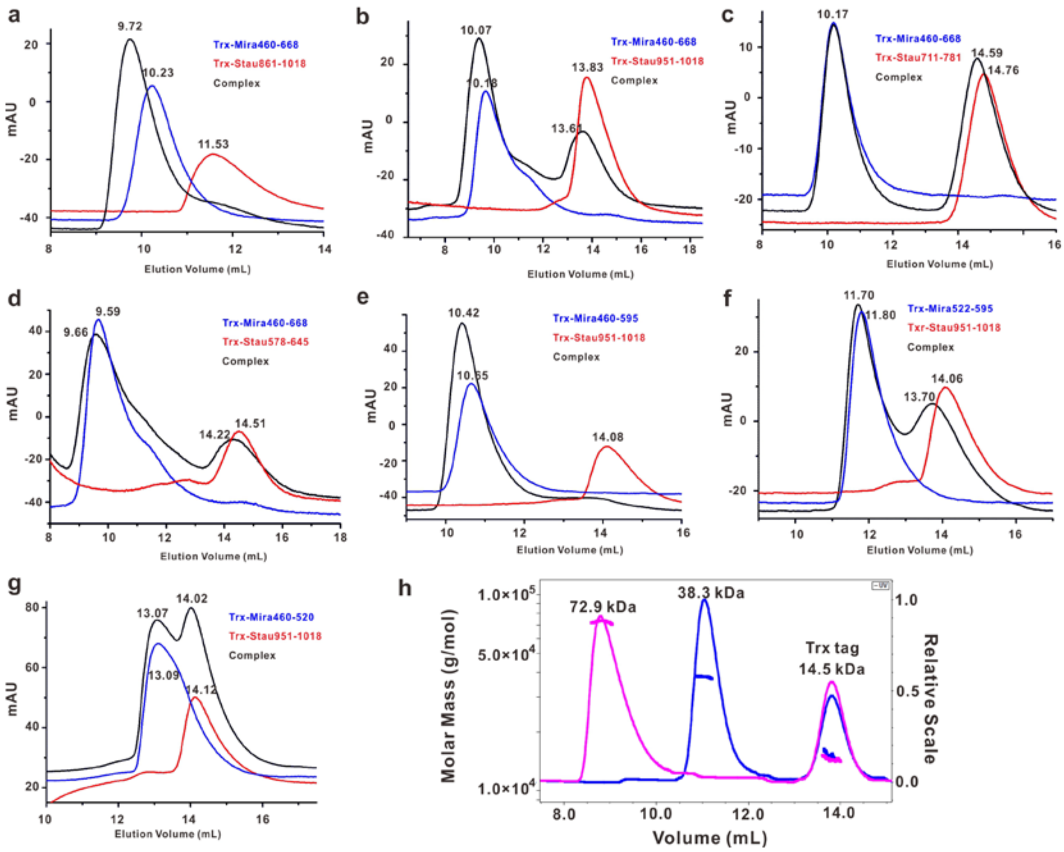


**Supplementary Figure 1.** Mapping the exact binding regions between Mira and Stau. (**a-g**) SEC profiles of various Mira and Stau fragments and their mixtures. The elution volumes of the peaks are indicated. (**h**) Static light scattering measurements of the molecular weights of Mira/Stau complexes. Blue: Mira514-595/Stau dsRBD5 (aa 951-1018) complex. The theoretical molecular weight of the 2:2 complex is 33.7 kD. Magenta: Mira514-595/Stau861-1018 complex. The theoretical molecular weights of the 2:2 and 2:4 complexes are 51.1 and 83.7 kD, respectively. Trx tags were cut from the fusion proteins and used as an internal standard.

Method: Protein samples (100 µl at a concentration of 20 µM, preequilibrated with the column buffer) were injected into an AKTA FPLC system with a Superose 12 10/300 GL column (GE Healthcare) with the column buffer of 50 mM Tris (pH 7.8), 100 mM NaCl, 1 mM DTT, and 1 mM EDTA. The chromatography system was coupled to a static light-scattering detector (miniDawn, Wyatt) and differential refractive index detector (Optilab, Wyatt). Data were analyzed with ASTRA 6 (Wyatt).

**Supplementary Figure 2**

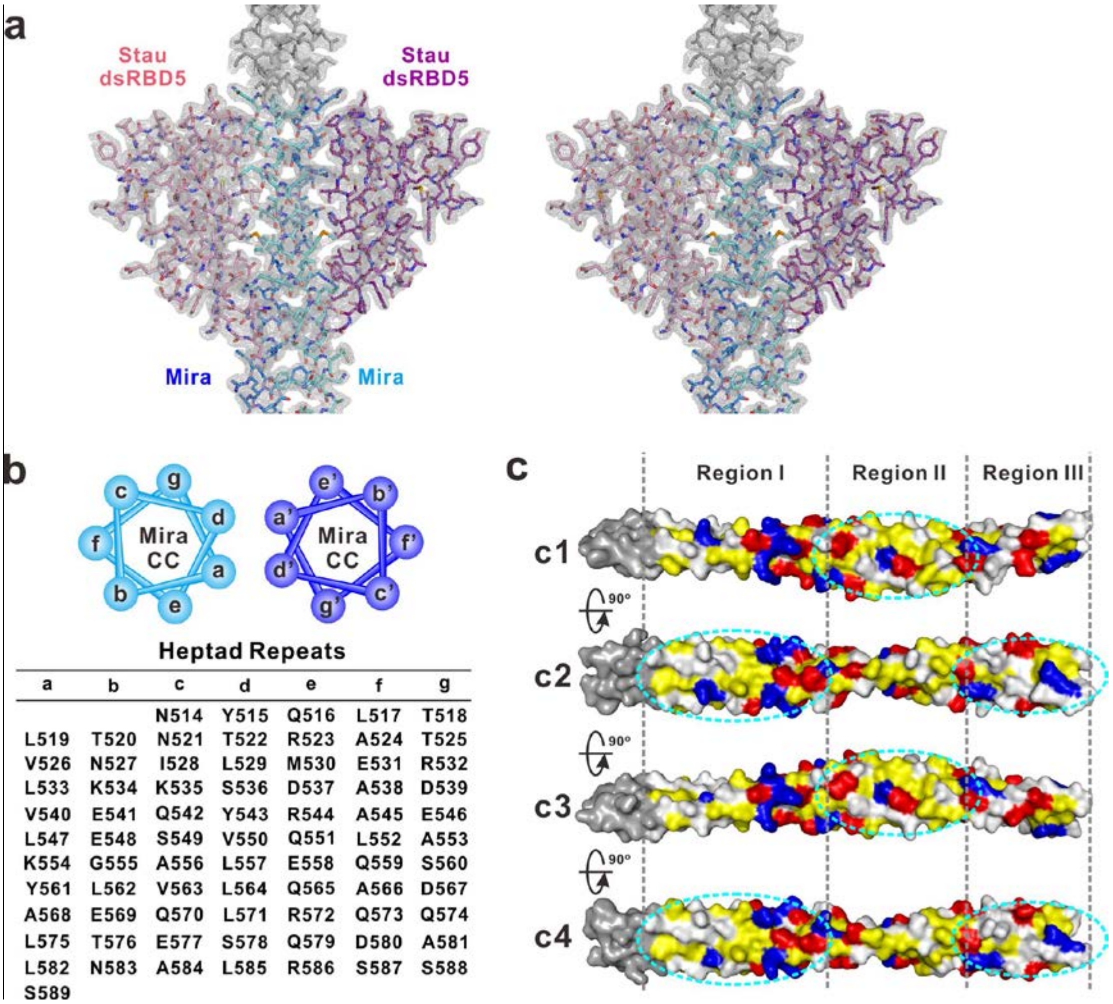


**Supplementary Figure 2.** The Mira/Stau complex structure. (**a**) A stereo image of the 2Fo-Fc map for the interaction between Mira514-595 and Stau dsRBD5 in the Miran/Stau structure at 1.49 Å contoured at 1.0 σ. (**b**) Helical wheels representation of the Mira514-595 coiled-coil dimer and the amino acid sequence of the heptad repeats. (**c**) Surface representation of Mira514-595 coiled-coil showing three potential target binding regions. In this drawing, the hydrophobic residues are in yellow, the positively charged residues in blue, the negatively charged residues in red, and the rest of the amino acids in gray. The orientation of Mira in **c1** is the same as that in Figure **2b**.

## Supplementary Figure 3

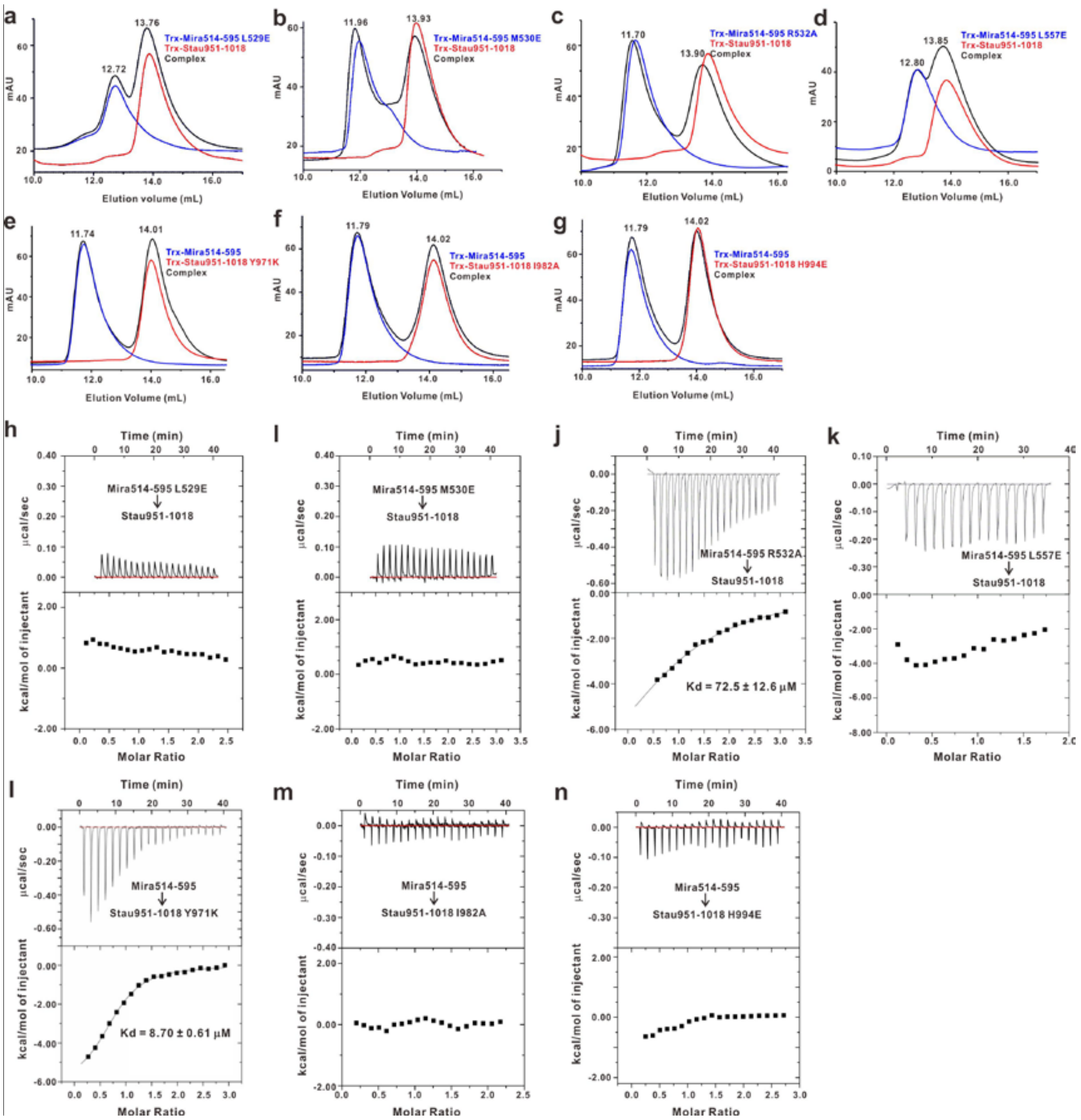

**Supplementary Figure 3.** Validation of the Mira514-595/Stau dsRBD5 complex structure by site-directed mutations. SEC (**a-g**) and ITC (**h-n**) analyses showing that the site-directed mutations (L529E$^{Mira}$, M530E$^{Mira}$, R532A$^{Mira}$, L557E$^{Mira}$, Y971K$^{Stau}$, I982A$^{Stau}$, and H994E$^{Stau}$) based on the crystal structure disrupted or impaired the interaction between Mira and Stau dsRBD5.

## Supplementary Figure 4

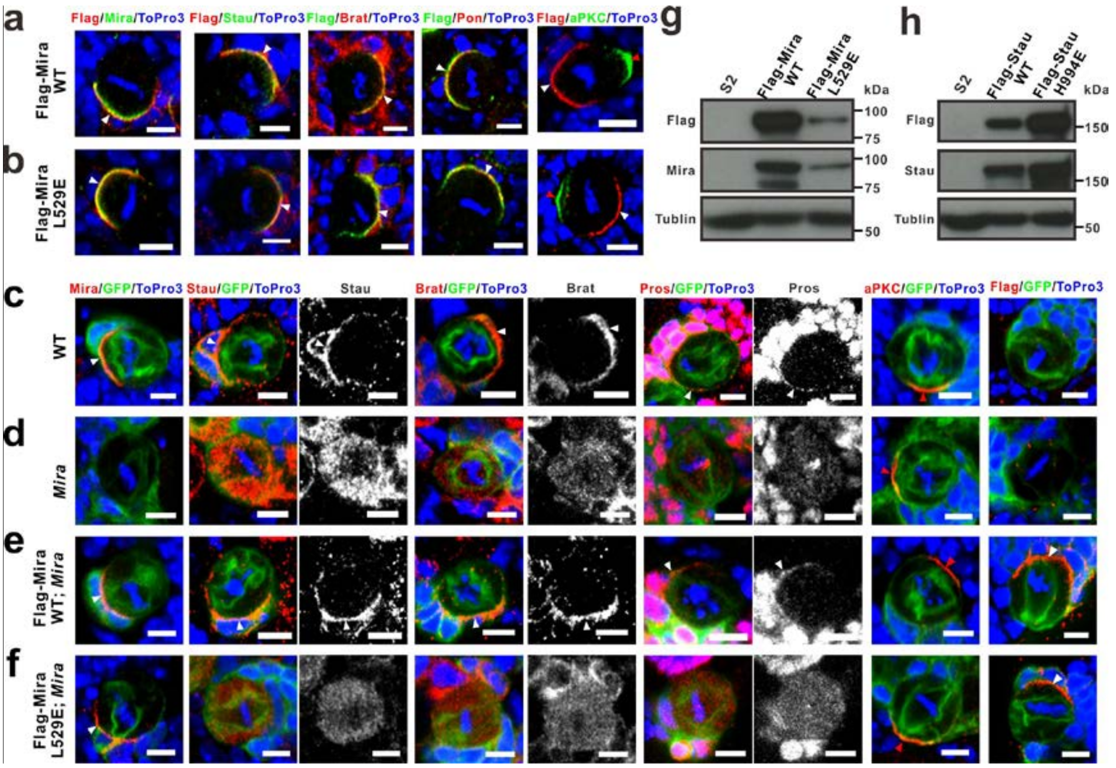


**Supplementary Figure 4.** Direct Mira-Stau interaction is required for Stau localization during the asymmetric divisions of *Drosophila* type I larval NBs. (**a**-**b**) Expressing Flag-Mira WT (**a**) or Flag-Mira L529E (**b**) in WT NBs (n=20 for each genotype) of larval brains driven by *insc-gal4*. ToPro-3 in blue. Flag-Mira WT and Flag-Mira L529E are asymmetrically localized. Stau, Brat, Pon and aPKC are normally localized. (**c**-**f**) Staining of various apical and basal proteins (red) in larval NBs (marked by GFP) generated by MARCM derived from WT (**c**), *Mira* mutant (**d**), *Mira* mutant expressing a *Flag-Mira WT* transgene (**e**), and *Mira* mutant expressing a *Flag-Mira L529E* transgene (**f**) (n=20 for each genotype). (**c**) Mira, Stau, Brat, Pros and aPKC are asymmetrically localized in WT NBs. (**d**) Mira is not detected in *Mira* mutant NBs, whereas Stau, Brat and Pros are cytoplasmic in *Mira* mutant NBs. aPKC is normally localized in a *Mira* mutant NB. (**e**) Mira (detected by anti-Mira antibody or anti-Flag antibody), Stau, Brat, Pros and aPKC are normally localized in *Mira* mutant NBs rescued with Flag-Mira WT. (**f**) In *Mira* mutant NBs rescued with Flag-Mira L529E, majority of NBs exhibit basal localization of Mira L529E. Stau, Brat and Pros remain in cytoplasm and aPKC is normally localized in these NBs. White arrowheads point to basal cortex whereas red arrowheads indicate apical cortex. Scale bars, 5 μm. (**g**-**h**) Western blot showing expression of Flag-Mira (**g**) or Flag-Stau (**h**) variants in *Drosophila* S2 cells. Uncropped blots are shown in Supplementary Fig. 6.

**Supplementary Figure 5**

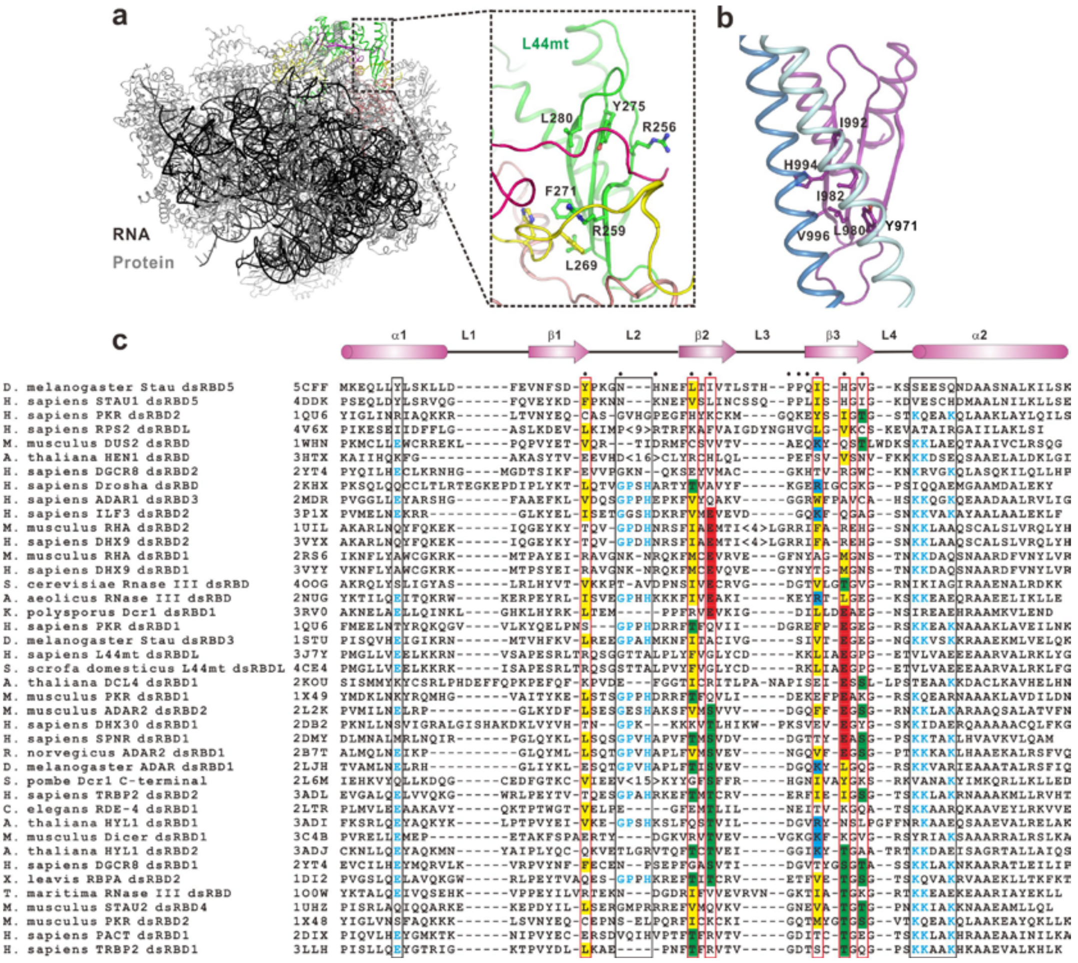


**Supplementary Figure 5.** The exposed β-sheet might be a common protein binding surface for dsRBDs. (**a**) The cryoelectron microscopy structure of human mitochondrial ribosome (PDB ID: 3J7Y) illustrating that the dsRBDL of L44mt might use the β-sheet face to interact with ribosomal proteins. The inset to the right shows the detailed interaction between dsRBDL (green) and various ribosomal proteins (magenta, yellow, and pink). The side chains of residues involved in the interaction are drawn in the stick model. The residues from the β-sheet of L44mt dsRBDL form two independent patches to interact with two ribosomal proteins. (**b**) The Mira/Stau dsRBD5 complex. The orientation of dsRBD5 is the same as L44mt dsRBDL in panel **a**. (**c**) Structure-based sequence alignment of *Drosophila* Stau dsRBD5 with all the dsRBDs and dsRBDLs deposited in PDB bank. Secondary structures of Stau dsRBD5 are shown at the top of the panel. The residues of Stau dsRBD5 involved in Mira binding are indicated with dots. The key residues on the β-sheet face of Stau dsRBD5 involved in Mira binding are highlighted with red boxes. Sequence consensus (≥20 %) of these key residues from different dsRBDs and dsRBDLs is highlighted, with the hydrophobic residues in yellow, the positively charged residues in blue, the negatively charged residues in red, and the Ser/Thr residues in green. Three dsRNA-binding regions are indicated by black boxes. The key residues for dsRNA-binding are highlighted in cyan.

**Supplementary Figure 6**

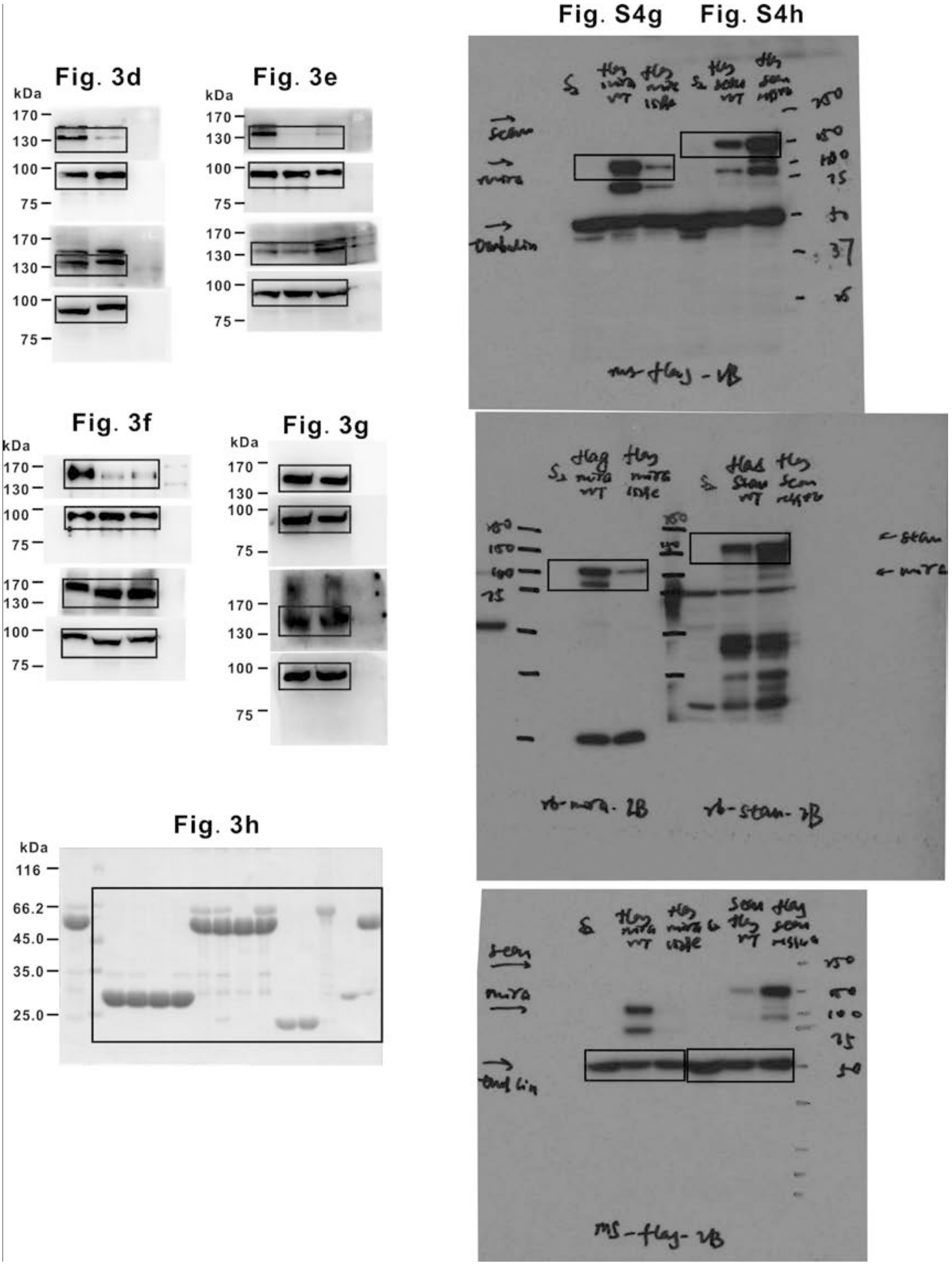


**Supplementary Figure 6.** Full uncropped figures of western blots and SDS-PAGE gels. Cropped regions are indicated with rectangles as appropriate.

.

**Supplementary Table 1. Primers for site-directed mutagenesis**

| Mutations | Forward primer (5'-3') | Reverse primer (5'-3') |
|---|---|---|
| Mira L529E | GTCAACATTGAGATGGAGCGT | ACGCTCCATCTCAATGTTGAC |
| Mira M530E | ATTCTGGAGGAGCGT | ACGCTCCTCCAGAAT |
| Mira R532A | CTGATGGAGGCCCTAAAGAAG | CTTCTTTAGGGCCTCCATCAG |
| Mira L557E | AAGGGAGCGGAGGAGCAGAGC | GCTCTGCTCCTCCGCTCCCTT |
| Stau Y971K | TCGGACAAGCCGAAAG | CTTTCGGCTTGTCCGA |
| Stau I982A | CTGACCGCCGTGACA | TGTCACGGCGGTCAG |
| Stau H994E | ATCTGCGAAGGCGTT | AACGCCTTCGCAGAT |
